# Effect of Handgrip Training in Extreme Heat on the Development of Handgrip Maximal Isometric Strength among Young Males

**DOI:** 10.3390/ijerph18105240

**Published:** 2021-05-14

**Authors:** Ignacio Bartolomé, Jesús Siquier-Coll, Mario Pérez-Quintero, María Concepción Robles-Gil, Diego Muñoz, Marcos Maynar-Mariño

**Affiliations:** 1 Department of Physiology, School of Sport Sciences, University of Extremadura, 10003 Cáceres, Spain; ignbs.1991@gmail.com (I.B.); marioperezquintero10@gmail.com (M.P.-Q.); mmaynar@unex.es (M.M.-M.); 2 Movement, Brain and Health Research Group (MOBhE), Center of Higher Education Alberta Giménez, Comillas Pontifical University, 07013 Palma de Mallorca, Spain; 3 Department of Didactics of Musical, Plastic and Corporal Expression, School of Teacher Training, University of Extremadura, 10003 Cáceres, Spain; mcroblesgil@gmail.com (M.C.R.-G.); diegomun@unex.es (D.M.)

**Keywords:** strength training, isometric strength, heat acclimation, performance, handgrip

## Abstract

The aim of this study was to evaluate the acute and adaptive effects of passive extreme heat (100 ± 3 °C) exposition in combination with a strength training protocol on maximal isometric handgrip strength. Fifty-four untrained male university students participated in this investigation. Twenty-nine formed the control group (NG) and 25 the heat-exposed group (HG). All the participants performed a 3-week isotonic handgrip strength training program twice a week with a training volume of 10 series of 10 repetitions with 45-s rest between series, per session. All the subjects only trained their right hand, leaving their left hand untrained. HG performed the same training protocol in hot (100 ± 3 °C) conditions in a dry sauna. Maximal isometric handgrip strength was evaluated each training day before and after the session. NG participants did not experience any modifications in either hand by the end of the study while HG increased maximal strength values in both hands (*p* < 0.05), decreased the difference between hands (*p* < 0.05), and recorded higher values than the controls in the trained (*p* < 0.05) and untrained (*p* < 0.01) hands after the intervention period. These changes were not accompanied by any modification in body composition in either group. The performance of a unilateral isotonic handgrip strength program in hot conditions during the three weeks induced an increase in maximal isometric handgrip strength in both hands without modifications to bodyweight or absolute body composition.

## 1. Introduction

In the context of sport performance, heat stress has been traditionally considered a critical issue by physicians and athletes, especially in endurance disciplines [1]. Thus, the exposure of humans to heat by using saunas or thermal chambers has been used for several decades as a strategy to increase heat tolerance as well as to decrease its negative side effects on human physical performance [2].

Heat, by itself, develops interesting physiological responses beyond these endurance effects and information has emerged over the last few years on the positive effect of muscle temperature on its mechanical and metabolic properties [3]. In addition, new ergogenic properties have recently been attributed to heat in the context of high-intensity physical performance [4] as well as in muscle anabolism [5]. Additionally, a recent review suggests that heat stress seems favorable for short-term, maximal performance [6].

High-intensity physical exercises generally lead to dramatic demands on the nervous and metabolic systems [7,8], as well as to high amounts of muscle damage; as occurs in the case of strength and resistance training, which are generally characterized by high indices of mechanical and metabolic stress [9].

In the context of strength, promising effects have been linked to heat in the last few years as it has been observed that tetanic force production can be increased after a rise in muscle temperature [10,11,12] mediated by a higher rate of actin and myosin coupling action [12] and by a heat-induced inhibition of pre- and post-synaptic action potentials [13]. These positive responses are commonly induced by repeated exposure to high environmental temperatures, which lead to a neuroprotective response in the central nervous system (CNS) accompanied by an increase in cognitive function under these conditions [14,15]. In this line of thought, several heat-induced adaptions have been observed in anabolism [5,16], substrate utilization [17], and even in submaximal force production among elderly individuals [16].

In the context of clinical strength training and evaluation, both researchers and physicians have considered the handgrip as an interesting and reliable tool for the assessment of functional strength [18] and prediction of sport performance and general health [19].

It has to be highlighted that in the literature in this field, the absence of a proper control group is a common limitation [2,20]. In order to overcome this problem, a control group of considerable size (*n* = 29) and of similar characteristics to the heat-exposed participants (see Table 1) was recruited for this study. Additionally, as far as the authors are aware, only three recent papers have used extreme heat (100 °C) to acclimate human subjects [21], but with different aims which go beyond this topic.

Therefore, this study aimed to evaluate three heat-induced responses: first, the acute effect of extreme heat (100 ± 3 °C) by itself or in combination with strength training on maximal isometric handgrip strength; second, the difference between unilateral isotonic handgrip strength and unilateral training in the heat (100 ± 3 °C) on maximal isometric handgrip strength, and third, the possible effects of heat in cross-adaption to unilateral handgrip strength.

## 2. Materials and Methods

Strength tests were programmed just before (in basal, rested conditions) and just after heat exposure to assess the acute (immediate) effect. As repeated exposure to heat increases thermal tolerance, daily evaluations of strength were designed to monitor the evolution of the effects of heat instead of applying a pre-post intervention evaluation.

Additionally, in order to isolate the effect of heat, the authors decided that participants should train just the right hand, using the left hand as a control parameter to avoid the muscular fatigue induced by handgrip training. For this purpose, two study groups were formed with both groups performing the same handgrip strength training protocol in two different thermal conditions. One group performed this protocol under normothermia (NG) and the other in hot conditions in the sauna (HG).

**Figure 1:**
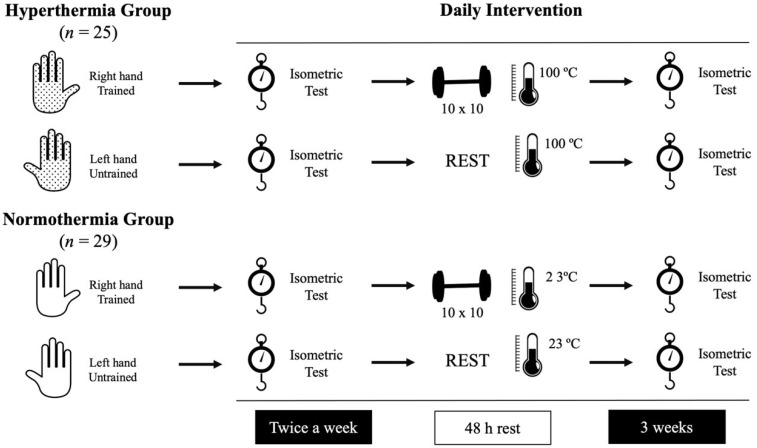
Representation of the daily intervention followed by the participants.

This experimental design was applied for three weeks, monitoring strength (basal and after the training sessions) in both groups daily. The first (day 1) and the last (day 6) sessions were used to evaluate the pre-post intervention differences. Figure 1 illustrates the experimental design.

### 2.1. Subjects

In order to meet the guidelines of Chow et al. 2017 [22], sample size was calculated and sixty male untrained university students were initially recruited. In the calculation process, the alpha risk was 5%, beta risk was 20%, correlation coefficient was 0.45, and possible estimated losses were 3%.

Thirty participants were randomly assigned to the normothermia group (NG, age: 21.75 ± 0.34 years; height: 1.75 ± 0.05 m; weight: 72.06 ± 7.46 kg; BMI: 23.01 ± 2.26; fitness: 5.15 ± 1.72 h/week) and thirty formed the hyperthermia group (HG, age: 21.23 ± 0.55 years; height: 1.76 ± 0.06 m; weight: 72.75 ± 6.89 kg; BMI: 23.88 ± 2.10; fitness: 4.78 ± 2.13 h/week). The randomization of the participants was carried out following the guidelines of Suresh 2011 [23], using a simple randomization. The randomization was performed by using the Random Allocation Software^®^ and was carried out by one of the authors before the beginning of the experimental period.

Five dropouts occurred in the HG and one NG participant could not complete the study. Dropouts were due to schedule incompatibilities, and information regarding these participants was not considered in the statistical analysis.

All the participants were recruited in the Sport Sciences School of the University of Extremadura by means of pamphlet distribution and voluntary informative talks.

All of them fulfilled the inclusion criteria: being healthy men without any kind of pathology or disease, not having suffered muscle or joint injuries or immobilizations in the previous 6 months, not having performed any kind of strength protocol or strength training in the previous 8 months, not having taken any supplementation, drug, or alcohol in the previous two months, and leading a healthy lifestyle: being physically active, and practicing endurance and recreative exercise three to five times per week. All participants were novel in strength training and none of them had previously performed any kind of strength training.

Each participant was previously informed about the study aims, techniques, and protocols before the beginning of the investigation, and all of them were asked to give their signed consent after accepting their voluntary participation.

This study was approved by the bioethics committee of the University of Extremadura (Register number: 32//2020) under the Helsinki Declaration ethic guidelines for human research of 1975, updated at the World Medical Assembly in Fortaleza 2013.

### 2.2. Procedures

#### 2.2.1. Health Security Criteria 

In order to ensure that all subjects were healthy and did not have any contraindication to take part in the study, a general medical evaluation was conducted with each participant, and they were assessed by a physician before the beginning of the experimental period. The medical exam consisted of a routine medical evaluation.

Additionally, during the heat exposure protocol in the sauna, body temperatures (internal—IT; external—ET) were monitored using an infrared thermometer (TAT 5000 “Exergen Temporal Scanner”, Exergen, Watertown, MA, USA) just before and after each series. If a participant reached 41 °C of internal body temperature, he was cooled using ice and cold water until IT was normalized (36 °C) and the physician evaluated him. Additionally, if a participant felt unable to complete the 10 min in the sauna, regardless of whether or not the IT reached 41 °C, his sauna session was stopped and he was immediately evaluated by the physician. No heat shocks were reported.

#### 2.2.2. Initial Evaluation

In the initial evaluation, each participant received a simple medical examination and completed the international physical activity questionnaire (IPAQ) [24]. They were then randomly assigned to the NG or HG.

#### 2.2.3. Strength Training Protocol

Each participant completed a total of six isotonic unilateral strength training sessions with a frequency of two sessions per week, with the total duration of the training program three weeks.

Since it has been reported in the literature for neural mechanisms to be predominant in the early phase of training [25,26], and to contribute principally to force production from the second training week [27], and for hypertrophic processes to start contributing from the third or fourth weeks [26,28], a three-week period duration was established in order to meet the specific effect of heat upon mechanisms of force production beyond structural hypertrophy.

Additionally, despite the fact that it has been widely reported in the literature that strength training can induce contralateral adaptions [29,30], the effect of extreme heat in this training-induced response is still unclear. For this reason, the training was performed only with the right hand, using the left hand as an intraindividual control parameter.

The training system consisted of 10 series of 10 isotonic repetitions of maximal manual grip with a handgrip clamping spring. The recovery time among series was fixed at 45 s.

#### 2.2.4. Heat Exposure Protocol

The HG performed the training protocol in a Finnish sauna (Harvia, C105S, Logix Combi control; 3 to 15 KW, Harvia, Muurame, Findland) using a dry protocol. The sauna temperature was fixed at 100 °C (100 ± 3 °C) with RH the same as in the laboratory conditions. In order to ensure that temperature and humidity were the same in all training sessions, after each sauna session the sauna was ventilated and reheated until the temperature reached 100 °C. Once the target temperature had been reached the participants entered and completed the 10 series. If they finished the training in less than 10 min, they remained in the sauna in resting conditions until the 10 min of exposure were completed. The total heat exposure time in the study was 60 min per participant, with a weekly volume of 20 min (10 min per session).

#### 2.2.5. Body Composition and Body Temperature Evaluations

Body composition measurements were performed by electrical bioimpedance (Body Composition Analyzer BF-350, Tanita Corp., Tokyo, Japan), and evaluations of each participant were made on days 1, 3, and 6 of the intervention and always followed the manufacturer’s guidelines. Bodyweight was evaluated daily, before and after each intervention.

Additionally, the body temperature was taken of each participant on their forehead (external temperature—ET) and on the right inner side of their mouth (internal temperature—IT), with the mouth completely closed. Temperature evaluation was carried out before and after each intervention using an infrared thermometer (TAT 5000 “Exergen Temporal Scanner”, Exergen, Watertown, MA, USA).

#### 2.2.6. Muscular Strength Evaluations

Maximal handgrip isometric strength was evaluated in both hands in all participants every day they trained in the intervention with tests before and after each training session.

The protocol used to evaluate the participants’ strength started with 15 repetitions in a warm-up phase using a soft foam ball, then each participant took the handgrip dynamometer (Takei A5401, Niigata City, Japan) and performed a maximal isometric strength test. This instrument has been previously validated and considered reliable for strength evaluation [31].

In order to ensure the reliability of the test, each participant had to perform the tests with their arm extended and their hand attached to their hip. A 4-s maximal voluntary contraction was fixed as a necessary requirement. The evaluations began with the right arm and after a rest period of 60 s, the same protocol was applied to the left arm. All evaluations were carried out in the morning, keeping each participant’s evaluation time constant throughout the study.

Once the pre-post session evaluations were completed, the fatigue index was calculated by subtracting the final (post-session) value from the respective initial (pre-session) one.

### 2.3. Statistical Analysis

IBM SPSS Statistics software, version 24 for Macintosh was used to perform the statistical analysis. The analysis consisted of a normality study using the Kolmogorov-Smirnov test. Once the normality of the data was rejected, the Wilcoxon test for paired samples was used to evaluate the intragroup differences between the different evaluation time points. Effect sizes were calculated by means of the Cohen’s R test for nonparametric data following the guidelines of Fritz et al. [32]. Additionally, the Mann–Whitney U test was used to determine differences among groups in each evaluation. A *p*-value of 0.05 was fixed to determine the limit of statistical significance.

## 3. Results

### 3.1. Body Composition

Body composition variables are presented in Table 1. No differences between groups were observed at any moment of the study. The NG did not experience any change during the intervention, however, the HG experienced a decrease in body lean mass percentage (*p* < 0.05; R = 0.21) and body water percentage (*p* < 0.05; R = 0.33) at the end of the study in comparison to the respective initial values.

### 3.2. Maximal Isometric Handgrip Strength

Table 2 presents the main results regarding the effect of the intervention program on handgrip strength, body temperature, and bodyweight variables. Results compare day 1 (pre-intervention) and day 6 (post-intervention) in both groups.

Regarding the pre-intervention results, no pre-post session differences were observed among the NG participants. In contrast, the HG participants experienced a post-session decrease in the trained hand (*p* < 0.01; R = 0.86) with unaltered values in the untrained one. Additionally, the HG participants experienced a greater increase in fatigue (FI) (*p* < 0.01) as well as a lower post-session difference between hands (*p* < 0.05) in comparison to their counterparts from the NG.

Regarding the responses to the whole training program, the NG did not show any training effect in either hand. An increase was observed, however, in the difference between hands after (post-training) the last session of the program (*p* < 0.05), which is reflected in a greater increment in the difference between hands in comparison to their respective values at the beginning of the study (*p* < 0.05; R = 0.65)

The HG experienced several changes in handgrip strength: pre-session values of isometric strength at the end of the intervention were significantly higher than pre-intervention ones in the trained hand (*p* < 0.05; R = 0.77). Interestingly, the isometric strength of the untrained hand also increased at the end of the intervention (*p* < 0.05; R = 0.81). Finally, the difference in strength between both hands in basal conditions decreased at the end of the study (*p* < 0.05; R = 0.68).

**Figure 2:**
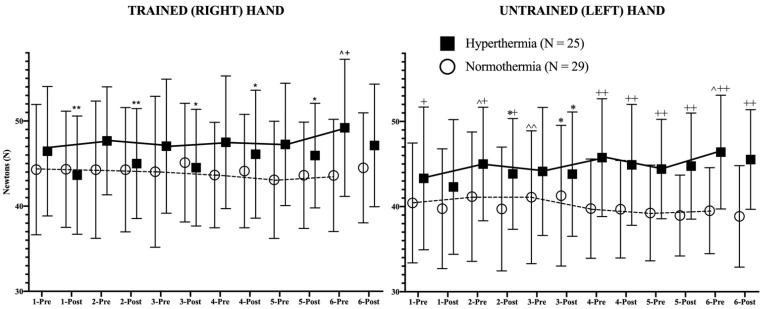
Strength evolution of both trained (right) and untrained (left) hands during the study. The continuous (hyperthermia) and the discontinuous (normothermia) lines represent the evolution of strength in basal (pre-exercise) conditions. Wilcoxon Test: Pre-post differences of each day in each group: * *p* < 0.05; ** *p* < 0.01; Pre-pre differences between consecutive days for each group: ^ *p* < 0.05; ^^ *p* < 0.01. Mann–Whitney U Test: Differences between groups at each time point: + *p* < 0.05; ++ *p* < 0.01.

In addition to these results, the daily evolution of the isometric strength pre- and post-session of both hands in both study groups is represented in Figure 2, revealing the statistical differences in strength between pre-post training sessions as well as the evolution of isometric strength in basal (pre-session) conditions between consecutive days.

Regarding the results of the trained hand in both groups, it has to be mentioned first that the trend line showed an opposite behavior pattern; while the HG showed a positive trend in strength, their counterparts experienced a negative evolution of strength during the study. Among the NG participants, no statistical changes can be observed in Figure 2. However, the HG experienced a highly significant (*p* < 0.01) decrease in isometric strength in hot conditions after the first two sessions (first week of training). From the third training session (second week of training) the decrease in strength after training in the heat was lower in statistical terms (*p* < 0.05). Finally, in the last session of the program, no post-session decline in strength could be observed.

In the case of the untrained hand, similar trends were observed. Although the NG experienced an increase (*p* < 0.01) in strength on the third day in basal (pre-session) strength in comparison to the previous values, the global strength trend was negative, obtaining lower values at the end of the intervention. The HG participants experienced a progressive increase in maximal isometric strength during the study, without pre- and post-session differences, with the exception of days 2 and 3, in which the post-training values decreased significantly (*p* < 0.05). The differences between days in pre-session (basal) conditions reached statistical significance on days 2 (*p* < 0.05), and 6 (*p* < 0.05). From the 4th day, the HG presented higher (*p* < 0.01) values than the NG in both pre-session and post-session conditions.

**Figure 3:**
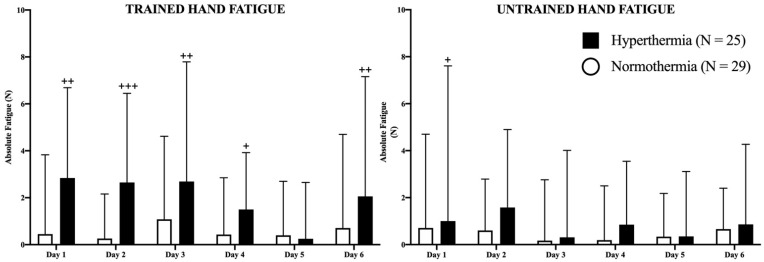
Representation of fatigue in both hands as well as the evolution of the differences between hand strength during the study. The filled (hyperthermia) and empty (normothermia) columns represent the evolution of strength in basal (pre-exercise) conditions. Mann–Whitney U Test: Differences between groups at each time point: + *p* < 0.05; ++ *p* < 0.01; +++ *p* < 0.001.

In Figure 3, absolute fatigue for each hand in both groups is represented in newtons (N). The HG presented higher values of fatigue in the trained hand on days 1 (*p* < 0.01), 2 (*p* < 0.001), 3 (*p* < 0.01), 4 (*p* < 0.05), and 6 (*p* < 0.01) than the NG, without changes between days in any study group. Regarding the untrained hand, the HG experienced higher values of fatigue than the NG only on the first day (*p* < 0.05). No more differences were observed in the study between consecutive days.

### 3.3. Body Temperature and Bodyweight Evolution

Table 2 shows information about IT and ET before and after sessions at the beginning (pre-intervention) and at the end (post-intervention) as well as the effect of these sessions on bodyweight loss. Regarding the differences in bodyweight, it can be observed in the table that none of the participants in the NG experienced any change in bodyweight either in terms of acute effect (differences between pre- and post-session) or in the long term (pre- and post-intervention). In the case of the HG, bodyweight did not change after the intervention in basal conditions; however, the performance of a handgrip training session in hyperthermic conditions led to a similar decrease in bodyweight compared to before entering the sauna both at the beginning (*p* < 0.01) and at the end (*p* < 0.01) of the investigation.

From the body temperature analysis, it can be observed in the table that the NG increased the levels of IT (*p* < 0.001) and ET (*p* < 0.05) after the training session at the beginning of the study. In the case of the final evaluation, ET, which increased (*p* < 0.01), was the only one affected by the training sessions. The HG increased (*p* < 0.001) both temperatures after the first session as well as the last session. Both temperatures after the first session were higher in the HG than in the NG. At the end of the intervention, the HG presented higher IT (*p* < 0.01) and ET (*p* < 0.001) than the NG after the final session.

**Figure 4:**
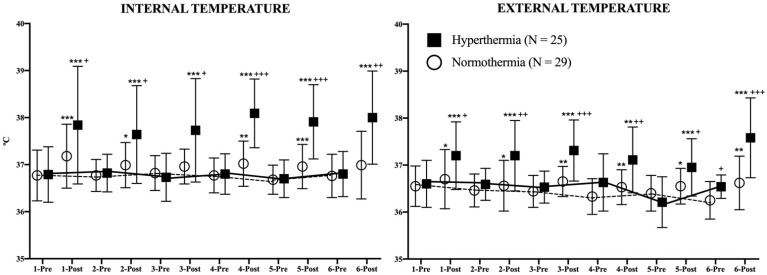
Evolution of internal and external temperatures during the study. The continuous (hyperthermia) and the discontinuous (normothermia) lines represent the evolution of strength in basal (pre-exercise) conditions. Wilcoxon Test: Pre-post differences of each day in each group: * *p* < 0.05; ** *p* < 0.01; *** *p* < 0.001. Mann–Whitney U Test: Differences between groups at each time point: + *p* < 0.05; ++ *p* < 0.01; +++ *p* < 0.001.

Figure 4 represents the evolution of both temperatures in both study groups during the study. For each day of the intervention, IT and ET were evaluated just before and immediately after each session. Post-session IT was higher among the HG participants in comparison to the NG (days 1–3: *p* < 0.05; days 4–5: *p* < 0.001; day 6: *p* < 0.01). In the analysis of the differences between pre-post session IT, it can be observed that HG increased its values every day (*p* < 0.001) while the NG did the same on days 1 (*p* < 0.001), 2 (*p* < 0.05), 4 (*p* < 0.01), and 5 (*p* < 0.001). No differences were observed in basal conditions in IT between consecutive days in either group.

ET was also affected in this study. The HG increased its post-session values every day (days 1–3 and 4–6: *p* < 0.001, day 5: *p* < 0.01) as well as the NG (days 1, 2, and 5: *p* < 0.05; days 3, 4, and 6: *p* < 0.01). The post-session ET values were higher in the HG in comparison to the NG in all evaluations (days 1 and 5: *p* < 0.05; days 2 and 4: *p* < 0.01; days 3 and 6: *p* < 0.001). Similar to IT, no differences were found in ET in basal conditions between consecutive days in either study group.

## 4. Discussion

First, the effect of heat exposure on body composition will be discussed. As can be observed in Table 2, no changes were observed among control participants, which suggests that the handgrip strength training alone did not provide a sufficient stimulus to induce morphological adaptions in three weeks. Among the HG participants, the performance of the same training program in hot conditions (100 ± 3 °C; 40 ± 2% RH) led to a decrease in body lean mass and body water percentages (*p* < 0.05). As total bodyweight was also maintained during the study the obtained results may suggest body re-composition. However, the results of this investigation do not coincide with previous reports, which documented a heat-induced increase in body water content [33]. The dissonance in the results could be explained by the short duration (3 weeks) and the short volume of total exposure to heat (a total of 60 min) in the acclimation program used in this study, as well as the absence of endurance training in the heat, an aspect which, as has been widely demonstrated, induces an increase in plasma volume and body water [2]. The other possible explanation is that although the manufacturer’s guidelines were followed in the body composition evaluation, the bioimpedance analysis has its own limitations for estimating body composition [34].

In all cases, bodyweight remained constant, which in combination with the low volume of muscle mass implied in the handgrip training, and considering the short duration of the program, make the idea of muscle hypertrophy unlikely, with the main causes of the strength gains probably being linked to the neural and metabolic responses [27], a factor which will be discussed below.

The use of a sauna has clear acute effects on body temperature and bodyweight loss, as shown in Table 2. Although the handgrip training sessions alone (among the NG participants) led to rises in body temperatures, indicating an exercise-induced increase in metabolic activity [35], the increases were less marked than among the heat-exposed participants. The HG showed considerably higher IT and ET values after all training sessions in the heat, a fact which has been widely documented [36], and which implies the trigger mechanism for heat thermoregulatory responses [2]. However, and as occurred among the NG participants, at the end of the study, basal ET and IT did not change in comparison with the initial values. Interestingly, basal ET was higher (*p* < 0.05) in the HG in comparison to the NG, with both groups starting with similar values.

The effect of heat on body water losses seems clear in this study, the HG participants decreased their bodyweight significantly after the sauna sessions, suggesting an acceleration of the thermoregulatory systems, in combination with the rises in body temperatures [37]. As has been previously indicated, the pre-session bodyweight of all participants was unaltered during the study, with the final values were similar to the initial ones.

In this study, both groups performed the same handgrip strength training (10 series of 10 repetitions with rest intervals of 45 s). As the number of participants in this study is considerable (NG: *n* = 29; GH: *n* = 25), the fact that control participants did not increase their strength values at the end of the program, while the heat-exposed participants did increase theirs, may prove a heat-induced effect in the investigation.

This effect could act by enhancing the exercise-induced adaptions to strength training. The fact that the NG did not increase their values could be due to the intensity of the training program. In this respect, it is well documented that 3 weeks is long enough to induce strength increments, mainly mediated by neuroendocrine and neuromotor responses [38]. So, the explanation of the results obtained by the control participants may be based on the insufficient intensity of the training program in young, healthy men. This idea is reinforced by the data in Figure 2, where constant values can be observed in pre-training sessions during the study in the NG. Additionally, considering the nature of the training program, which was mainly metabolic training, it should be considered that it may have induced more transference to muscular endurance than to maximal neuromuscular isometric strength, the variable analyzed in this research.

Interestingly, the fact that the HG increased their values can be explained by several theories, with heat playing a critical role in all of them. Firstly, if the fatigue indices (FI) of both groups are compared (Figure 4), it can be observed that the HG participants experienced greater fatigue after all training sessions with the exception of session 5, especially in the right (trained) hand. Interestingly, although statistical significance was not reached, a greater trend is present in left (untrained) hand values, if these values are compared with the untrained FI of the NG, suggesting possible heat-induced fatigue, especially in the first four sessions.

In this respect, it has been reported that heat exposure can affect the central nervous system, decreasing the motor drive to the skeletal muscle [7], which may explain the greater FI among the HG and the lower values in a highly neural task such as maximal voluntary isometric contraction.

This may indicate two responses. The first is the response to training in the heat performed by the right hand. Considering that the control participants did not experience any fatigue or supercompensation responses, and taking into account the motor unit recruitment principles, it is plausible that the handgrip training alone did not provide a sufficient stimulus to recruit fast-twitch (FT) fibers or to induce strength increases, while in hot conditions, fatigue of the slow-twitch (ST) fibers would be induced by neural [39], circulatory [40], or metabolic [41] factors, leading to a greater rate of recruitment of the FT fibers. Additionally, as the HG had greater initial values of strength in both hands than the controls, this hypothesis become more interesting for the explanation of the heat-induced strength gains.

In this respect, if most ST fibers were fatigued, the recruitment rate of FT fibers may have increased, implying that more muscle mass was involved in the training, facilitating more neural stimuli to be released to recruit more fibers, leading to the neuromuscular adaptions [42]. It is well-documented and accepted that strength training induces an increase in the electrical activity of the neuromuscular system [30], and evidence exists on growth of motoneurons induced by exercise, an adaption that underlies the enhanced electric responses [43]. In this regard, an anabolic effect of heat has been reported [16,44,45] which may contribute to the growth of motoneurons as well as to the increments in short-term muscular strength.

Linked to this idea are the responses of the non-trained hand, which showed increases in maximal voluntary strength after the heat program without training. This result was reported when unilateral training obtains contralateral strength gains [46]. As with short-term training, the main adaptions underlying these gains are linked to the nervous system [29]. Interestingly, control participants did not increase their maximal handgrip strength while the heat-exposed participants increased their values, so heat may also play an important role in exercise-induced adaptions.

The explanation of these results is linked to several heat-induced responses, and considering muscle hypertrophy as unlikely, the strength gains could be based on neural, endocrine, or metabolic development. It is known that heat stress acts directly on the central nervous system because the thermoreceptors are connected to the hypothalamus, the central axis of the thermoregulatory responses [47]. Furthermore, the central nervous system is extremely sensitive to thermal perturbations and it has been reported that heat exposure induces a thermoprotective response, making the nervous system more resistant to heat [15]. This mechanism could contribute to strength development making the motor cortex and motoneurons more resistant to heat and, consequently, less fatigable in hot conditions. It is interesting that after session 4 the FIs were markedly lower in comparison to the first sessions in the heat. Additionally, it is well known that heat exposure increases two strong anabolic pathways, the mammalian target of rapamycin (mTOR), a critical signaler peptide in the initiation of translation of protein synthesis [48]. The second anabolic pathway is the overexpression of the heat shock proteins (HSPs) which regulate peptide folding and which contribute to the repairing of the denaturized proteins [49]. In this regard, it is known that strength training may modulate the levels of HSPs27 [50]. Both pathways develop a net increase in protein turnover, decreasing protein catabolism and increasing peptide anabolism as well. As motoneurons grow after strength training [43], both these pathways could contribute to greater nerve tissue synthesis which induces greater neural growth, and consequently greater neural-induced strength development. Although this hypothesis seems interesting, more research is required in this field to discover the specific responses of the nervous system to the combination of heat and strength training stimulus.

Regarding skeletal muscles, it is well known that protein synthesis and peptide adaptions increase after just one strength training session [43]. Although net muscle hypertrophy is unlikely in 3 weeks, enzymatic and metabolic responses linked to protein synthesis may occur in this period of time, contributing to strength development.

Improvements have been reported in maximal anaerobic performance after heat treatment protocols [6], the physiological background of these gains being an increase in the rate of anaerobic production of energy with its respective increase in enzymatic activity [51,52,53], an increase in muscle fiber electrical conduction velocity [51,54,55], or a greater contractile ability of muscles through faster sequestration of Ca^2+^ with or without changes in the activities of ATPases [56]. Interestingly, these increases in anaerobic performance have been accompanied sometimes by greater rates of fatigue (as occurred in the first sessions of this study) [57,58]. Additionally, two important muscle receptors of muscle tension and metabolism (P2X and TRPV1) are temperature sensitive [59] and develop important afferent reflex responses in perceived exertion and fatigue. It is known that their activity is influenced by temperature, with lower rates at higher muscle temperatures [60]. So, it is plausible that the adaption to heat may affect the activity of these receptors, decreasing the fatigue-inducing muscle afferent reflexes [61], leading to higher muscular performance. Furthermore, it has been reported that heat treatment is an interesting adjunct in the recovery of strength and range of movement [62].

Finally, although ample evidence exists of a favorable effect of heat exposure and acclimation in muscular strength, the effect of a whole-body, passive heat acclimation to extreme heat in handgrip strength has not been evaluated yet.

The performance of a unilateral isotonic handgrip strength program in hot conditions of three weeks of duration induced an increase in the maximal isometric handgrip strength in both hands without modifications in bodyweight or absolute body composition. Although the mechanisms underlying these gains are still unknown, the heat exposure developed a positive effect because control participants did not experience any change after the intervention. Several nervous and metabolic hypotheses could explain the obtained results, as the hypothesis of muscle hypertrophy is unlikely.

However, several important limitations still exist in this field, these will have to be highlighted and considered in future investigations. The first one is linked to the type of strength training performed; resistance or maximal strength of greater muscles groups, or multiarticular exercises or whole-body exercises linked to sport, fitness, or rehabilitation have not been evaluated, since the obtained results in this work could not be extrapolated to these fields. In future research, basic strength/resistance exercise should be analyzed.

The second one concerns the compensatory responses to training, which have not been evaluated; the last strength evaluations were carried out on the last training day. Some evaluations should be performed after a reasonable compensatory period of time, once the training program has been concluded. Additionally, larger duration training programs should also be considered.

Finally, another important limitation to consider is the absence of gender differences. As all participants in this work were males, information regarding women is still unknown, and further research is required. In order to meet the human responses to extreme heat, women should also be evaluated in future investigations.

For these reasons, the positive outcomes of this study must be interpreted with caution, as knowledge in this field is still scarce and further research is required to contrast the obtained results.

Since extreme heat has not already been considered a strength ergogenic, these results are novel, and the outcomes of this work should be interpreted carefully, however, and considering these limitations, the use of passive exposure to extreme heat in combination with strength training could be of interest in several professional fields linked to strength development, such as clinical training, where heavy resistance loads could compromise muscle or joint integrity.

In all cases, further research is required in this field to contrast the positive outcomes linked to heat, as well as to discover the specific pathways underlying these responses.

## 5. Conclusions

The performance of a three-week duration unilateral isotonic handgrip strength program in hot conditions induced an increase in the maximal isometric handgrip strength in both trained and untrained hands, without modifications to bodyweight or absolute body composition among heated participants, and the values of normothermia participants were unaffected.

Additionally, greater fatigue indexes were observed among these participants, in comparison to their respective counterparts.

In light of the obtained results, the use of sauna baths with extreme dry heat may be an interesting tool to increase strength in the short term. However, despite the interest in these results, several limitations still exist. Therefore, the outcomes of this work should be interpreted carefully.

## Figures and Tables

**Table 1:** Body composition variables of the participants during the study.

		Normothermia Group (NG; *n* = 29)	Hyperthermia Group (HG; *n* = 25)
Day 1	Bodyweight (Kg)	72.06 ± 7.46	72.75 ± 6.89
	Body Mass Index	23.01 ± 2.26	23.88 ± 2.10
	Basal Metabolism (Kcal/day)	1811.18 ± 121.57	1806.09 ± 110.36
	Body Fat Mass (%)	13.77 ± 4.00	13.65 ± 4.38
	Body Lean Mass (%)	86.37 ± 4.01	85.80 ± 6.19
	Body Water (%)	63.20 ± 2.93	63.30 ± 3.15
Day 3	Bodyweight (Kg)	71.31 ± 9.47	72.33 ± 6.85
	Body Mass Index	23.31 ± 2.08	23.70 ± 2.02
	Basal Metabolism (Kcal/day)	1818.13 ± 105.37	1766.90 ± 192.44
	Body Fat Mass (%)	13.95 ± 3.92	13.69 ± 4.04
	Body Lean Mass (%)	86.05 ± 3.93	85.82 ± 4.16
	Body Water (%)	86.05 ± 3.93	61.93 ± 6.24
Day 6	Bodyweight (Kg)	71.97 ± 7.14	72.12 ± 6.28
	Body Mass Index	23.30 ± 2.34	24.40 ± 2.05
	Basal Metabolism (Kcal/day)	1805.86 ± 105.35	1845.92 ± 100.57
	Body Fat Mass (%)	14.32 ± 4.04	14.95 ± 4.51
	Body Lean Mass (%)	85.28 ± 4.11	84.82 ± 4.58 ^♦,R1^
	Body Water (%)	62.15 ± 3.27	61.49 ± 4.66 ^^,^^♦,R2^

NG: Normothermia group; SG: Sauna group. Wilcoxon Test: Differences between consecutive evaluations for each group: ^ *p* < 0.05. Differences between evaluations 1 and 6: ♦ *p* < 0.05. Effect size: ^R1^ = 0.21; ^R2^ = 0.33.

**Table 2:** Maximal isometric handgrip strength during the study.

	Normothermia Group (NG; *n* = 29)						Hyperthermia Group (HG; *n* = 25)					
	Pre-Intervention			Post-Intervention			Pre-Intervention			Post-Intervention		
	Pre-Session	Post-Session	Diff.	Pre-Session	Post-Session	Diff.	Pre-Session	Post-Session	Diff.	Pre-Session	Post-Session	Diff.
Trained Hand (N)	44.29 ± 7.65	44.33 ± 6.82	0.45 ± 3.38	43.61 ± 6.59	44.50 ± 6.46	0.71 ± 3.99	46.56 ± 7.60	43.64 ± 6.94	−2.84 ± 3.85	49.20 ± 8.05 ^+,^^♦^^,R1^	47.13 ± 7.20	−2.06 ± 5.10
Untrained Hand (N)	40.43 ± 7.04	39.75 ± 7.03	−0.71 ± 3.99	39.52 ± 5.05	38.85 ± 5.96	−0.66 ± 1.74	43.31 ± 8.38	42.31 ± 7.91	−1.00 ± 6.61	46.40 ± 6.68 ^++,^^♦^^,R2^	45.53 ± 5.85 ^++^	−0.86 ± 3.41
Hands Diff. (N)	3.95 ± 4.45	4.41 ± 4.35	0.46 ± 0.10	3.64 ± 2.89	5.47 ± 2.71 *	1.83 ± 0.18 ^♦^^,R4^	3.19 ± 6.24	1.33 ± 5.73	−1.86 ± 0.51	2.80 ± 2.51 ^♦^^,R3^	1.60 ± 3.20 ^++^	−1.20 ± 0.69 ^++^
I Temp (°C)	36.77 ± 0.54	37.18 ± 0.68 ***	0.40 ± 0.59	36.76 ± 0.46	36.99 ± 0.72	0.23 ± 0.70	36.79 ± 0.59	37.84 ± 1.25 ***^+^	1.05 ± 1.25 ^++^	36.80 ± 0.48	38.00 ± 0.99 ***,^++^	1.19 ± 0.83 ^+++^
E Temp (°C)	36.55 ± 0.43	36.70 ± 0.63 *	0.14 ± 0.54	36.25 ± 0.40	36.62 ± 0.57 **	0.37 ± 0.55	36.60 ± 0.50	37.20 ± 0.72 ***^+^	0.60 ± 0.87	36.54 ± 0.25 ^+^	37.58 ± 0.85 ***,^+++^	0.91 ± 0.86 ^+^
Bodyweight (Kg)	72.06 ± 7.46	72.07 ± 7.47	0.01 ± 0.23	71.97 ± 7.14	71.89 ± 7.17	−0.08 ± 0.25	72.41 ± 6.92	72.28 ± 6.89 **	−0.12 ± 0.50 ^+++^	72.12 ± 6.28	72.28 ± 6.64 **	0.16 ± 0.15 ^+^

NG: Normothermia group; SG: Sauna group. Diff: Difference between pre and post values of each hand in each evaluation; Hands Diff.: Difference between right and left hands in each evaluation; I Temp: Internal temperature; E Temp: External temperature. Wilcoxon Test: Pre-post differences of each day in each group: * *p* < 0.05; ** *p* < 0.01; *** *p* < 0.001. Pre-pre differences between days 1 and 6: ♦ *p* < 0.05; Mann–Whitney U Test: Differences between groups at each time point: ^+^ *p* < 0.05; ^++^ *p* < 0.01; ^+++^ *p* < 0.001. Effect size: ^R1^ = 0.77; ^R2^ = 0.81; ^R3^ = 0.68; ^R4^ = 0.65.

## Data Availability

Not Applicable.
